# Protective Role of the Toll-Like Receptor 5 Agonist KMRC011 against Murine Colitis Induced by *Citrobacter rodentium* and Dextran Sulfate Sodium

**DOI:** 10.4014/jmb.2209.09048

**Published:** 2022-11-15

**Authors:** Jun-Young Kim, Sun-Min Seo, Han-Woong Kim, Woo-Jong Lee, Yang-Kyu Choi

**Affiliations:** 1 Department of Laboratory Animal Medicine, College of Veterinary Medicine, Konkuk University, Seoul 05029, Republic of Korea; 2 GC Biopharma Corporation, Gyeonggi-do, 16924, Republic of Korea; 3 Regenerative Dental Medicine Institute, Hysensbio, Gyeonggi-do, 13814, Republic of Korea; 4 CONNEXT Co. Ltd, Daegu, 41061, Republic of Korea

**Keywords:** Ulcerative colitis, toll-like receptor 5 agonist, KMRC011, NF-kB signaling pathway

## Abstract

This study aimed to identify the therapeutic ability of a novel toll-like receptor (TLR) 5 agonist, KMRC011, on ulcerative colitis induced by *Citrobacter rodentium* and dextran sulfate sodium in a C57BL/6N mouse model. Ulcerative colitis was induced in the mice by the oral administration of 1% dextran sulfate sodium in sterile drinking water for seven days *ad libitum*, followed by *C. rodentium* infection on the seventh day by intra-gastric administration (DSS-CT group). KMRC011 was administered intramuscularly at both 24 h and 15 min before (Treatment 1 group), and at both 15 min and 24 h after (Treatment 2 group) the *C. rodentium* infection. The length of the large intestine and histopathological counts were significantly greater and mucosal thickness was significantly thinner in the Treatment 1 group compared to the DSS-CT and Treatment 2 groups. *Il-6* and *Il-10* mRNA expression levels were upregulated, while *Ifn-γ* and *Tnf-α* mRNA expression levels were significantly downregulated in the Treatment 1 group, compared to the DSS-CT group. NF-κB p65 expression level was elevated due to ulcerative colitis in the DSS-CT group, but was significantly downregulated in the Treatment 1 group. Overall, KMRC011 showed protective effects against murine colitis by inhibiting NF-κB signaling.

## Introduction

Inflammatory bowel diseases (IBDs) such as Crohn’s disease and ulcerative colitis are characterized by colonic lesions and neutrophil infiltration, followed by ulceration and necrosis of the gastrointestinal tract [1]. Several molecular signaling pathways were associated with intestinal inflammation; however, an inflammatory signaling pathway may originate specifically from a connection between the intestinal commensal bacterium and the host mucosa [2]. A deeper understanding of the mechanisms causing chronic disruption and dysregulation of the gastrointestinal tract mucosal immune system is still lacking [3]. However, its pathogenesis may involve environmental factors, inheritance, abnormal immunology and infection [4], and induction of proinflammatory cytokine release by malfunctioning of pattern recognition receptors [5].

Many animal models, including spontaneous and inducible colitis models and genetically engineered and adoptive transfer models [6, 7], have been used to understand the factors involved in IBD that can aid in the development of therapeutics [8]. Murine colitis is often induced by chemical agents because of their ease in usage and in controlling disease severity [8]. Trinitrobenzene sulfonic acid (TNBS) and dextran sulfate sodium (DSS) have been commonly used to induce colitis in the past two decades [9]. Previous papers reported that TNBS induced symptoms similar to human Crohn’s disease and DSS induced symptoms similar to human ulcerative colitis in the murine model [10]. Water-soluble DSS (40-50 kDa) is usually administered at a 2 to 5% concentration to induce IBD [11].

*Citrobacter rodentium* (formerly *Citrobacter freundii* biotype 4280) is a gram-negative bacterium that infects laboratory mice. It is transmitted via the fecal-oral route and forms attaching and effacing (A/E) lesions in the gastrointestinal tract [12, 13]. The infected mice exhibited hyperplasia of colonic epithelial cells and disruption of colonic mucosa [14]. Enteropathogenic *Escherichia coli* and enterohemorrhagic *E. coli* also form A/E lesions but are poorly pathogenic in the mouse gastrointestinal epithelium. Induction of A/E lesion formation by *C. rodentium* causes goblet cell depletion, hyperplasia of crypt cells, and inflammatory cell infiltration via colonic barrier disruption [15, 16].

KMRC011 is a partially modified version of toll-like receptor (TLR) 5 agonist, derived from *Salmonella enterica* flagellin. To make the KMRC011, 34 amino acid residues in entolimod (previously known as CBLB502) were selectively eliminated to avoid possible immune response [17]. Ulcerative colitis is caused by the uncontrolled actions of nuclear factor kappa B (NF-κB) signaling pathway and proinflammatory cytokines such as TNF-α, and IL-1β, causing intestinal tissue damage [18]. KMRC011 showed radio-protective effects against 11 Gy total body irradiation in C57BL/6N mice by reducing apoptosis, upregulating DNA-repair genes, and regulating the NF-κB signaling pathway [19]. CBLB502 successfully decreased intestinal damage by altering the NF-κB signaling pathway and TNF-α level in TNBS-induced colitis [20]. In this study, a combination of DSS and *C. rodentium* was used to induce ulcerative colitis in C57BL/6N mice, followed by an evaluation of KMRC011’s protective role in ameliorating intestinal damages caused by ulcerative colitis.

## Materials and Methods

### Animal

Six-week-old female C57BL/6N mice (18-20 g) were purchased from Koatech (Republic of Korea). The mice were acclimatized for a week and bred in the animal facility at the College of Veterinary Medicine, Konkuk University. Animals were housed in sterile polycarbonate cages with a stainless steel lid in a pathogen-free environment with a 12-h light/12-h dark cycle and a temperature of 22 ± 2°C with 50 ± 10% humidity. The mice were given sterilized food, water, and wooden bedding. All procedures were approved (KU19111) and conducted in accordance with the guidelines of the Konkuk University Institutional Animal Care and Use Committee.

### Preparation of Bacterial Strain and DSS

*Citrobacter rodentium* strain DBS 100 (ATCC 51459) was cultured on Luria-Bertani (LB) agar and incubated overnight at 37°C. A single colony was then collected and cultured in LB broth on a shaking incubator overnight at 37°C. The cultured LB broth was centrifuged at 1,200 g for 10 minutes and washed three times with sterilized phosphate buffer saline (PBS). Then, pelleted bacteria were re-suspended in fresh LB broth (0.2 ml) to yield a cell density of 1.6 × 10^8^ CFUs (colony forming units). The CFU/ml was determined by plating serially diluted bacterial suspensions. DSS (MP Biomedicals, USA), having a molecular weight of 36-50 kDa, was dissolved in sterile distilled water (DW) at 1% concentration and administered orally *ad libitum* for a week.

### KMRC011 Administration

KMRC011 was manufactured and provided by the Korea Institute of Industrial Technology (Republic of Korea). The purity of KMRC011 was determined to be 100% by using size-exclusion high-performance liquid chromatography, and quality control was performed by Advanced Protein Technologies Corp. (Republic of Korea).

### Experimental Design

The mice were divided into 4 groups as follows (each group *n* = 6): PBS group (DW *ad libitum*, LB broth via intragastric gavage and PBS treatment), DSS-CT group (1% DSS *ad libitum*, *C. rodentium* infection via intragastric gavage and PBS treatment), Treatment 1 group (1% DSS *ad libitum*, *C. rodentium* infection via intragastric gavage, and 0.05 mg/kg KMRC011 treatment both 24 h and 15 min before *C. rodentium* infection) and Treatment 2 group (1% DSS *ad libitum*, *C. rodentium* infection via intragastric gavage, and 0.05 mg/kg KMRC011 treatment both 15 min and 24 h after *C. rodentium* infection). The mice were pretreated with 1% DSS or DW for 7 days. On the seventh day, the mice were infected with 1.6 × 10^8^ CFU of *C. rodentium* via oral gavage after 12 h of fasting. KMRC011 was diluted with PBS and injected intramuscularly twice at 0.05 mg/kg either before or after the *C. rodentium* infection. The mice were euthanized on the 17^th^ day using diethyl ether (Fig. S1). The spleen was removed, weighed in sterile conditions, and stored at -70°C. The large intestine (from the cecum to anus) was removed and the length was measured with a ruler. One part of the colon was removed and fixed in 10% neutral buffered formalin for histopathological analysis. The other part of the colon was immersed in liquid nitrogen and stored at -70°C for further studies.

### H&E Staining and Histopathological Analysis of Colitis

Both mid and distal colon was immersed in 10% buffered formalin and processed routinely for paraffin sectioning. Paraffinized samples were cut into 4-μm-thick sections and placed on a slide glass. The samples were stained with hematoxylin and eosin (H&E) following the standard protocol. The samples were examined using a BX51 light microscope (Olympus, Japan) and images were captured using DP71 software (Olympus). Mucosal thickness, mucosal inflammation, sub-mucosal inflammation, and goblet cell number per high-power field (magnification, x400) were analyzed by two independent researchers under blinded conditions.

### Quantitative Real-Time Polymerase Chain Reaction (RT-PCR) Analysis

Total RNA was extracted from the colon using TRIzol reagent (Ambion). The cDNA was synthesized by using a mixture of oligo-dT, dNTP, RNase OUT^tm^, 5X First Strand Buffer, 0.1 M DTT, M-MLV, and total RNA. Quantitative real-time polymerase chain reaction was carried using CFX96 real-time system (Bio-Rad, USA) and iQ SYBR Green Supermix kit (Bio-Rad) following the manufacturer’s instructions. The annealing temperature was 58°C, with 39 cycles of amplification. The RT-PCR primers used are shown in Table S1. The expression of each gene was normalized to that of the housekeeping gene β-actin. The threshold cycle of each gene was measured as the cycle number at which the fluorescent signal of the reaction crosses the threshold. Samples were analyzed in triplicate and mean values were plotted.

### Western Blot Analysis

Protein was extracted from the colon (20-25 μg) using tissue-protein extraction reagent (Thermo Scientific, USA). The concentration of protein was measured using the BCA^tm^ protein assay kit (Thermo Scientific), followed by boiling in 4X sample buffer at 95°C for 5 minutes. Before loading, equal amounts of the protein were electrophoresed on an 8% SDS-polyacrylamide gel and transferred onto nitrocellulose membranes (Immobilon-P nitrocellulose membranes, USA). The membranes were blocked with 5% non-fat dry skim milk in TTBS for 1 h at room temperature and incubated overnight with monoclonal antibodies against β-actin (1:2000, Santa Cruz Biotechnology, USA) and NF-κB p65 (1:1000, Santa Cruz Biotechnology) at 4°C. Then, the membranes were washed with TTBS and incubated with rabbit anti-mouse IgG-HRP secondary antibodies (1:2000, Santa Cruz Biotechnology). The blots were visualized using a Clarity Western ECL kit (Bio-Rad). The band intensities were quantified using ImageQuant LAS 4000 (GE Healthcare, England) and Multi-Gauge software (Fujifilm) and normalized to the intensity of β-actin.

### Statistical Analysis

Data were reported as the mean values ± standard deviations. Statistically significant differences between experimental groups were evaluated by the one-way analysis of variance (ANOVA) with post-hoc Tukey HSD test using GraphPad Prism 7.04 (GraphPad Software). The value of *p* < 0.05 was determined to be statistically significant.

## Results

### Pre-Treatment of KMRC011 Ameliorated the Decrease of Large Intestine Length and Mucosal Thickness

**Fig. 1:**
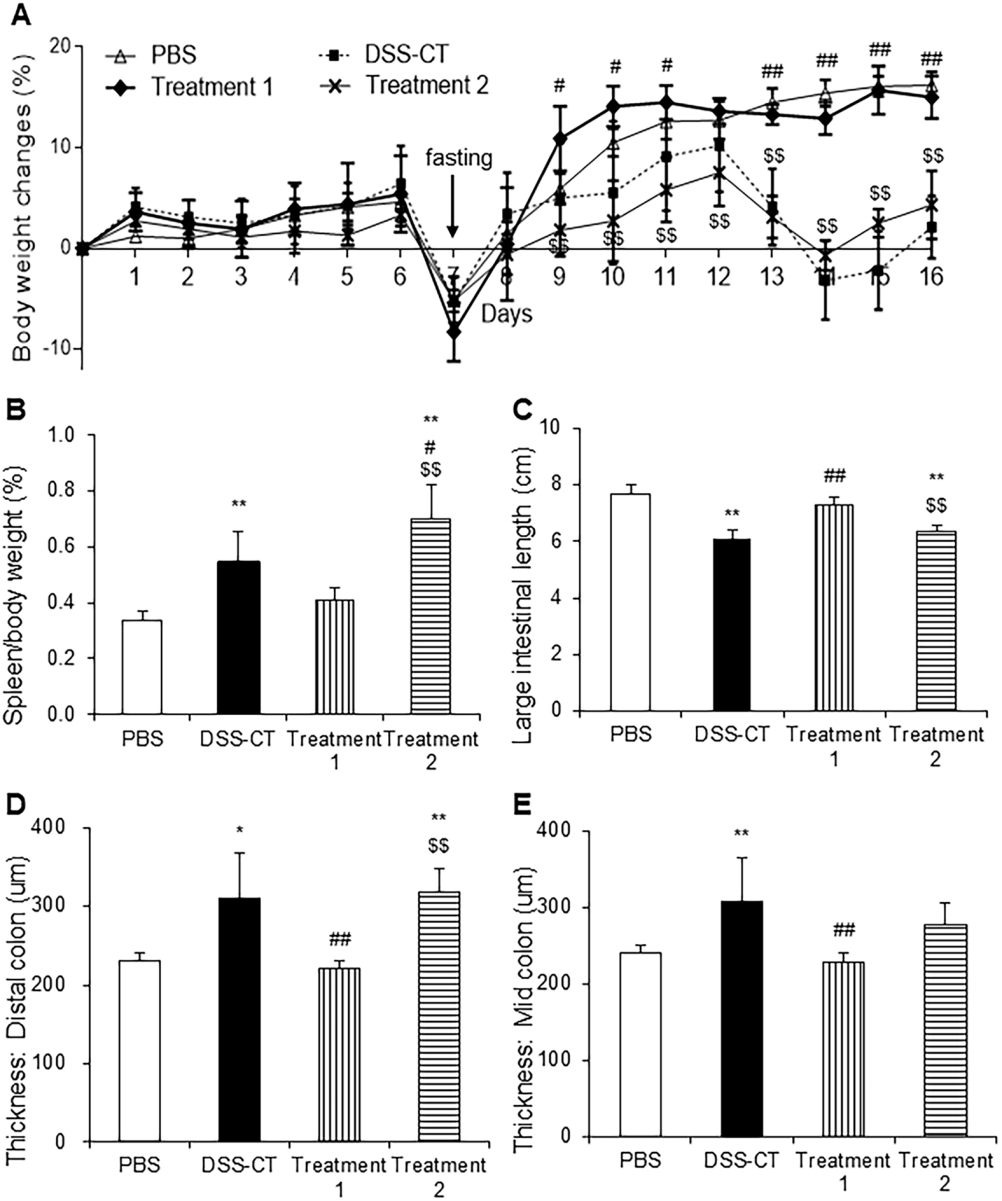
Effect of KMRC011 on body and spleen weight, intestinal length, and thickness of colon. Body weight changes (**A**) and spleen to body weight ratio (**B**) were expressed as percentages. (**C**) Length of entire large intestine from the cecum to anus was measured with a ruler. Mucosal thickness of the distal colon (**D**) and mid colon (**E**) were measured by 2 independent researchers. Data are presented as means ± standard deviation (SD). **p < 0.01 compared to the PBS group; ^#^*p* < 0.05 and ^##^*p* < 0.01 compared to the DSS-CT group; ^$^*p* < 0.05 and ^$$^*p* < 0.01 compared to the Treatment 1 group.

**Fig. 2:**
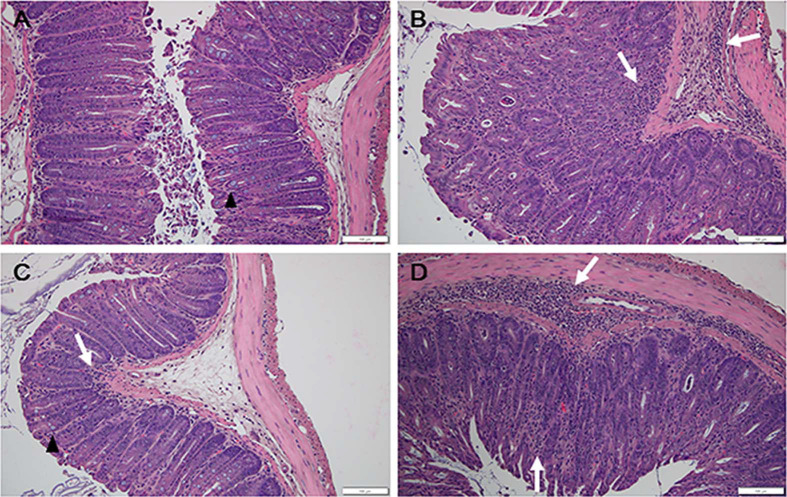
Representative images of histopathological lesions in the distal colon of PBS (A), DSS-CT (B), Treatment 1 (C), and Treatment 2 (D) group mice. Total intestinal thickness was increased in DSS-CT and Treatment 2 group mice. Goblet cells (black arrowheads) and inflammatory cell infiltration (white arrows) were observed in the colon. H&E stain. Scale bar = 100 um.

Body weight (%) of all groups increased slightly for the initial six days and dropped on day seven due to fasting prior to *C. rodentium* infection. Following infection, the body weight of mice from the PBS and Treatment 1 groups increased gradually till the end of the study, but those of mice from the DSS-CT and Treatment 2 groups increased only till day 12, and decreased thereafter. Body weight of Treatment 1 group was significantly (*p* < 0.05) higher than that of the DSS-CT and Treatment 2 groups (Fig. 1A). The spleen to body weight ratios (%) of the DSS-CT, Treatment 1, and Treatment 2 groups were significantly (*p* < 0.01) higher than that of the PBS group regardless of KMRC011 treatment. However, Treatment 1 group showed a significant (*p* < 0.05) decrease in the spleen to body weight ratio compared to the Treatment 2 groups (Fig. 1B). The length of the entire large intestine in the DSS-CT and Treatment 2 groups was significantly (*p* < 0.01) lesser than that in the PBS group (Fig. 1C). However, Treatment 1 group showed a significant (*p* < 0.01) increase in the length of the large intestine compared to the DSS-CT and Treatment 2 groups (Fig. 1C). Mucosal thickness of the colon was measured and compared to examine the disruption and hyperplasia of the colon using H&E staining. Mucosal thickness in Treatment 1 group was similar to that in the PBS group and significantly (*p* < 0.01) lesser in both distal (Figs. 1D and 2) and mid colon (Fig. 1E) compared to that in the DSS-CT and Treatment 2 groups.

### Pre-Treatment of KMRC011 Ameliorated Histopathological Lesion Formation in the Colon

**Fig. 3:**
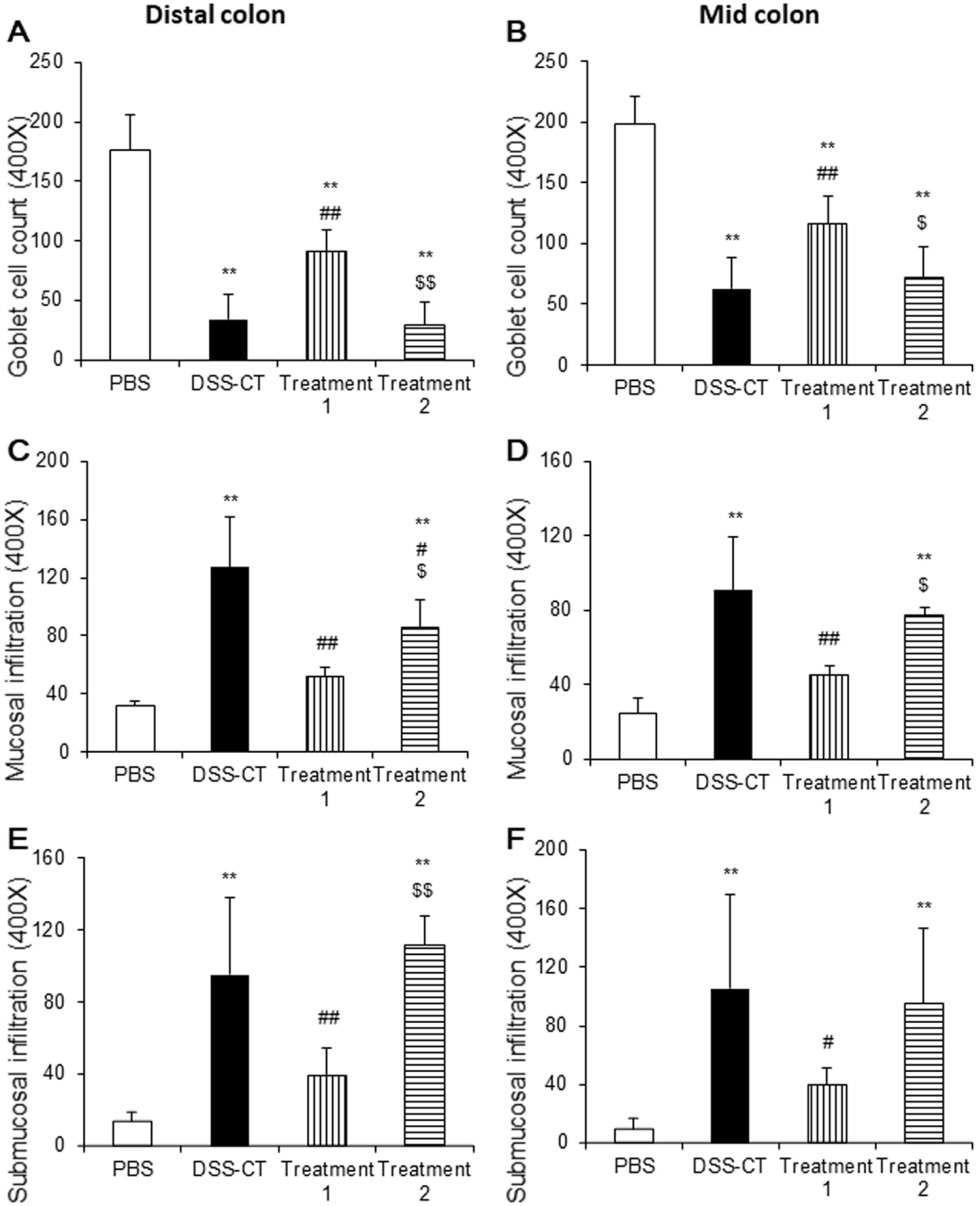
Effect of KMRC011 on the number of goblet cells and inflammatory cells in the colon. The number of goblet cells (**A, B**) and inflammatory cells infiltrated in the mucosa (**C, D**) and submucosa (**E, F**) of the distal (**A, C, E**) and mid (**B, D, F**) colon were counted by 2 independent researchers. Data are presented as means ± standard deviation (SD). **p < 0.01 compared to the PBS group; ^#^*p* < 0.05 and ^##^*p* < 0.01 compared to the DSS-CT group; ^$^*p* < 0.05 and ^$$^*p* < 0.01 compared to the treatment 1 group.

Histopathological lesions were evaluated by counting goblet cells and inflammatory cells in the colon by two investigators under blinded conditions. Goblet cell numbers in the ulcerative colitis groups were significantly (*p* < 0.01) lower compared to those in the PBS group regardless of KMRC011 treatment. However, Treatment 1 group had significantly (*p* < 0.05) higher goblet cell number compared to the DSS-CT and Treatment 2 groups in both the distal (Figs. 2C and 3A) and mid colon (Fig. 3B). The number of mucosal inflammatory cells in the ulcerative colitis groups were higher compared to the PBS group regardless of KMRC011 treatment. However, Treatment 1 group had significantly (*p* < 0.05) lower number of mucosal inflammatory cells in both the distal (Figs. 2C and 3C) and mid colon (Fig. 3D) compared to the DSS-CT and Treatment 2 groups. The number of submucosal inflammatory cells in the ulcerative colitis groups were significantly (*p* < 0.01) higher than in the PBS group regardless of KMRC011 treatment. However, their numbers in Treatment 1 group were significantly (*p* < 0.05) lower than that in the DSS-CT or Treatment 2 groups in both the distal (Fig. 3E) and mid colon (Fig. 3F).

### Pre-Treatment of KMRC011 Suppressed the Increase of Proinflammatory Cytokine mRNA Levels

**Fig. 4:**
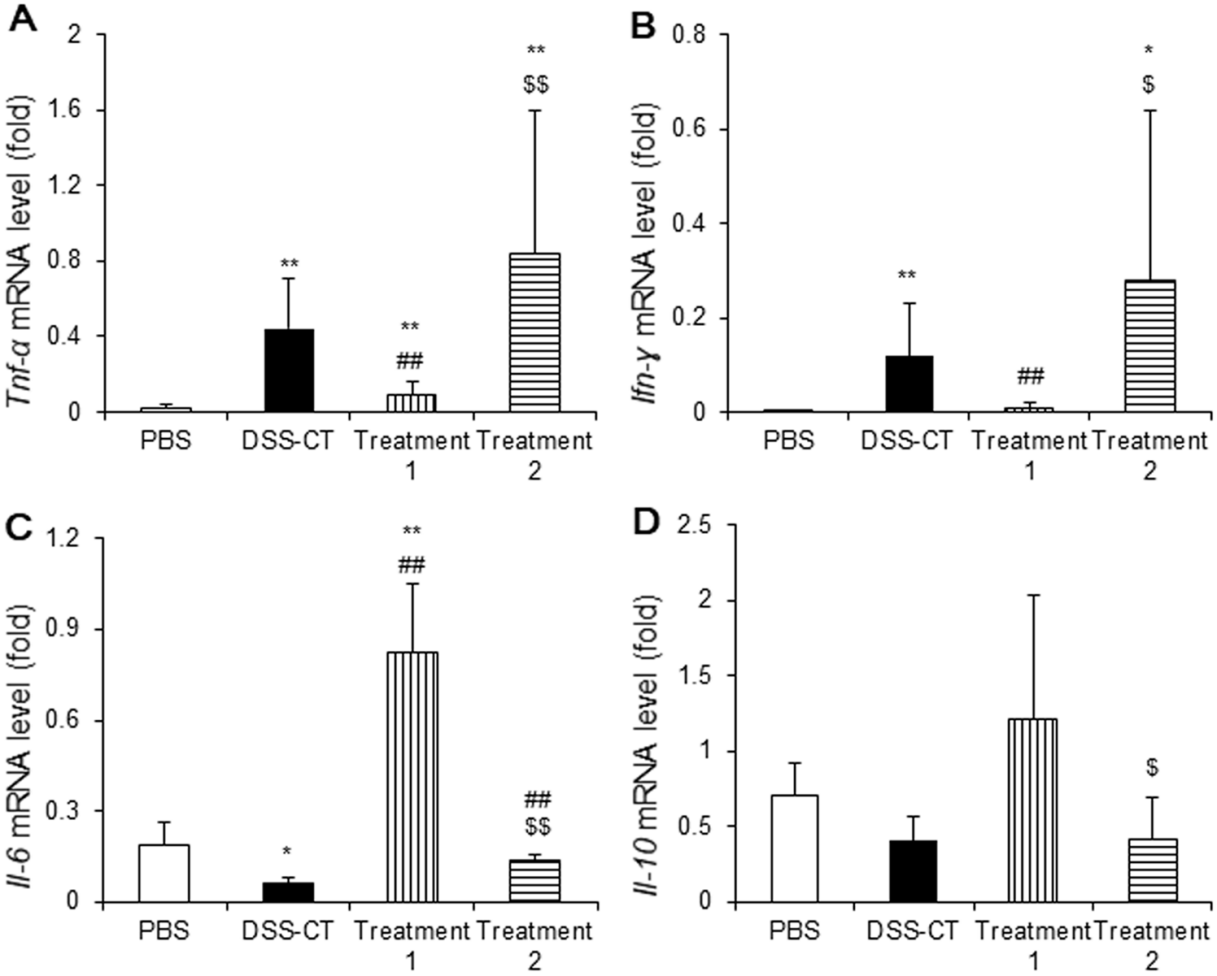
Effect of KMRC011 on the expression level of cytokines in the colon. The expression level of *Tnf-α* (**A**), *Ifn-γ* (**B**), *Il-6* (**C**) and *Il-10* (**D**) were measured using quantitative real-time polymerase chain reaction analysis. Data are presented as means ± standard deviation (SD). *p < 0.05 and **p < 0.01 compared to the PBS group; ^#^*p* < 0.05 compared to the DSS-CT group; ^$^*p* < 0.05 and ^$$^*p* < 0.01 compared to the Treatment 1 group.

Cytokines and chemokines have critical roles in modulation of inflammation, phagocytosis, and tissue injury. We examined the mRNA expression levels of *Tnf-α*, *Ifn-γ*, *Il-6*, and *Il-10* in the colon using RT-PCR. *Tnf-α* mRNA expression levels were significantly (*p* < 0.01) higher in all ulcerative colitis groups compared to the PBS group (Fig. 4A). However, Treatment 1 group showed significantly (*p* < 0.01) lower *Tnf-α* mRNA levels compared to the DSS-CT and Treatment 2 groups (Fig. 4A). *Ifn-γ* mRNA expression levels were significantly (*p* < 0.05) higher in the DSS-CT and Treatment 2 groups compared to the PBS group (Fig. 4B). However, Treatment 1 group showed a significant (*p* < 0.05) decrease in *Ifn-γ* mRNA level compared to the DSS-CT and Treatment 2 groups (Fig. 4B). In contrast, *Il-6* mRNA expression levels were significantly elevated (*p* < 0.01) in the Treatment 1 group compared to the other three groups (Fig. 4C). The DSS-CT group showed significantly (*p* < 0.05) lower *Il-6* mRNA expression levels than the PBS group (Fig. 4C). *Il-10* mRNA expression levels were increased in the Treatment 1 group compared to the PBS and DSS-CT groups, but no significant difference was observed (Fig. 4D).

### Pre-Treatment with KMRC011 Inhibits NF-κB Signaling

**Fig. 5:**
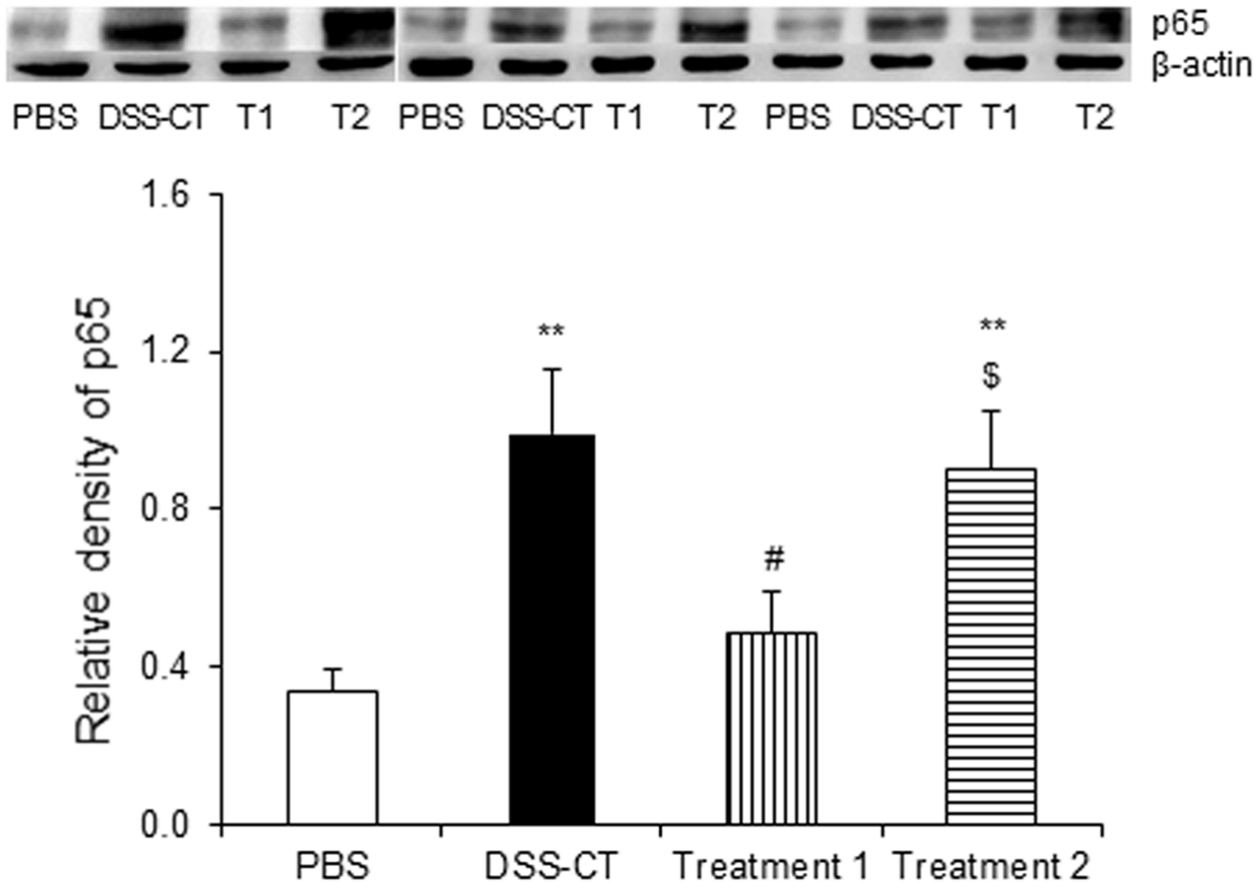
Effect of KMRC011 on the NF-κB p65 levels in the colon. Expression levels of the proteins were measured by Western blotting. The bands were quantified, and their expression was normalized to β-actin expression. Data are presented as means ± standard deviation (SD). **p < 0.01 compared to the PBS group; ^#^*p* < 0.05 compared to the DSS-CT group; ^$^*p* < 0.05 compared to the Treatment 1 group.

Western blot analysis was performed examine the changes in NF-κB p65 expression levels in the colon following KMRC011 treatment. NF-κB p65 protein expression levels were significantly higher in the DSS-CT and Treatment 2 groups than in the PBS group (*p* < 0.01). Treatment 1 group showed significantly lower (*p* < 0.05) NF-κB p65 expression levels than the DSS-CT and Treatment 2 groups (Fig. 5), but no statistical difference was observed compared from the PBS group (Fig. 5).

## Discussion

Chemically induced IBD models, such as TNBS- and DSS-induced IBD models, are widely used as they allow the easy manipulation of disease severity [8, 9]. *C. rodentium* forms A/E lesions in the intestine; these disrupt epithelial cells, leading to ulcerative colitis [16]. In our previous study, a murine model of ulcerative colitis induced by a combination of low-dose DSS and *C. rodentium* was established [21]. In the present study, the effectiveness of KMRC011 treatment against ulcerative colitis was examined to investigate its potential as a new therapeutic alternative.

The shortened length of the large intestine is an indirect indicator of mucosal inflammation [10]. The spleen to body weight ratio were significantly lower and the length of the entire large intestine was significantly longer in the Treatment 1 group than in the DSS-CT and Treatment 2 groups. This suggested that KMRC011 pre-treatment prevented the hypertrophy of the spleen and disruption of the colonic epithelial barrier. The damage was limited to the mucosal epithelial lining exhibiting features such as ulceration, mucosal infiltration, and hyperplasia of lymphoid tissue [22]. In the histopathological analysis, colonic morphology disruption was observed in the DSS-CT and Treatment 2 groups (data not shown). Significant thickening of the mucosa due to hyperplasia, goblet cell loss, and increased mucosal and sub-mucosal inflammatory cell infiltration were observed in the DSS-CT and Treatment 2 groups, compared to the PBS and Treatment 1 groups. This result is consistent with the previous colitis studies on goblet cell depletion, hyperplasia of the mucosa, increased mucosal infiltration and ulceration [12, 23]. Our data exhibited that KMRC011 ameliorated mucosal epithelial damage caused by ulcerative colitis.

IFN-γ is known to accelerate the damage to large intestinal epithelial cells [24]. Further, TNF-α concentration is increased in ulcerative colitis [25]. These proinflammatory cytokines are known to induce cytotoxicity, cellular proliferation, and regulation of innate immunity, which exacerbate the damage to the large intestine [26]. The anti-inflammatory cytokine IL-10 is produced by Th2 helper cells and B cells [27]. This is understandable, as ulcerative colitis is characterized by the immune response of Th2 cells [28]. In this study, the DSS-CT group showed significantly increased *Tnf-α* and *Ifn-γ* mRNA expression and decreased *Il-6* and *Il-10* mRNA expression compared to the PBS group. This phenomenon was a characteristic of *C. rodentium*- and DSS-induced colitis in our previous report [21]. Previous studies, however, have demonstrated that IL-6 level was elevated in a mouse model of DSS-induced ulcerative colitis [29] and blood of IBD patients [30]. It is quite conceivable that IL-6 can play both a pro-inflammatory and anti-inflammatory role during the colitis progress depending on the mouse models [31]. In the Treatment 1 group, the expression levels of proinflammatory cytokines such as *Tnf-α* and *Ifn-γ* were significantly decreased; however, the expression of inflammatory cytokines such as *Il-6* and *Il-10* were significantly increased compared to the DSS-CT group. This result showed that KMRC011 pre-treatment had protective effects against the progression of ulcerative colitis.

NF-κB signaling levels were increased in the mononuclear cells of the lamina propria of IBD human colonic biopsy samples [32] and colonic mucosa [33], indicating that NF-κB regulates the inflammatory response. Hence, inhibition of NF-κB is thought to be a key mechanism in decreasing colonic damage. NF-κB inhibitors such as infliximab have been used to confirm anti-inflammatory effects on *Trichuris muris*-induced chronic murine colitis [34, 35]. CBLB502, a slightly modified version of KMRC011 [17], significantly downregulates the TLR1, 2, 3, 4, 6, 7, 8, and 9 mRNA expression levels and had therapeutic effect in TNBC-induced colitis model by altering the NF-κB signaling pathway [20]. The TLR4 inhibitor naringenin decreases the NF-κB p65 level, which decreases colonic damage [36]. This study also showed that NF-κB p65 protein expression levels were significantly downregulated in the Treatment 1 group compared to the DSS-CT and Treatment 2 groups, indicating that KMRC011 pretreatment decreases the NF-κB p65 protein level, decreasing colonic damage. These findings represent that KMRC011 showed protective effects against murine colitis by inhibiting NF-κB signaling. Therefore, our data suggest that KMRC011 have the possible clinical benefit of preventive treatment of ulcerative colitis.

In summary, KMRC011 pre-treatment successfully reduced the degree of DSS-induced ulcerative colitis. This was proven by the greater length of the large intestine, higher number of goblet cells, thinner mucosal thickness, and lower number of infiltrating inflammatory cells. Moreover, consistent with histopathological lesions, downregulation of the components of the NF-κB signaling pathway and reduced inflammatory response were also observed in the KMRC011 pre-treatment group. Therefore, KMRC011 can be developed as an alternative protective agent against ulcerative colitis.

## Supplemental Materials

Supplementary data for this paper are available on-line only at http://jmb.or.kr.
