# Supplementary material for: Protective Role of the Toll-Like Receptor 5 Agonist KMRC011 against Murine Colitis Induced by Citrobacter rodentium and Dextran Sulfate Sodium
Source: J Microbiol Biotechnol. 2022 Nov 15;33(1):35–42. doi: 10.4014/jmb.2209.09048 (PMC9895994; doi:10.4014/jmb.2209.09048)
Supplement: Supplementary file 1 [file jmb-33-1-35-supple.pdf]

J. Microbiol. Biotechnol.
https://doi.org/10.4014/jmb.2209.09048

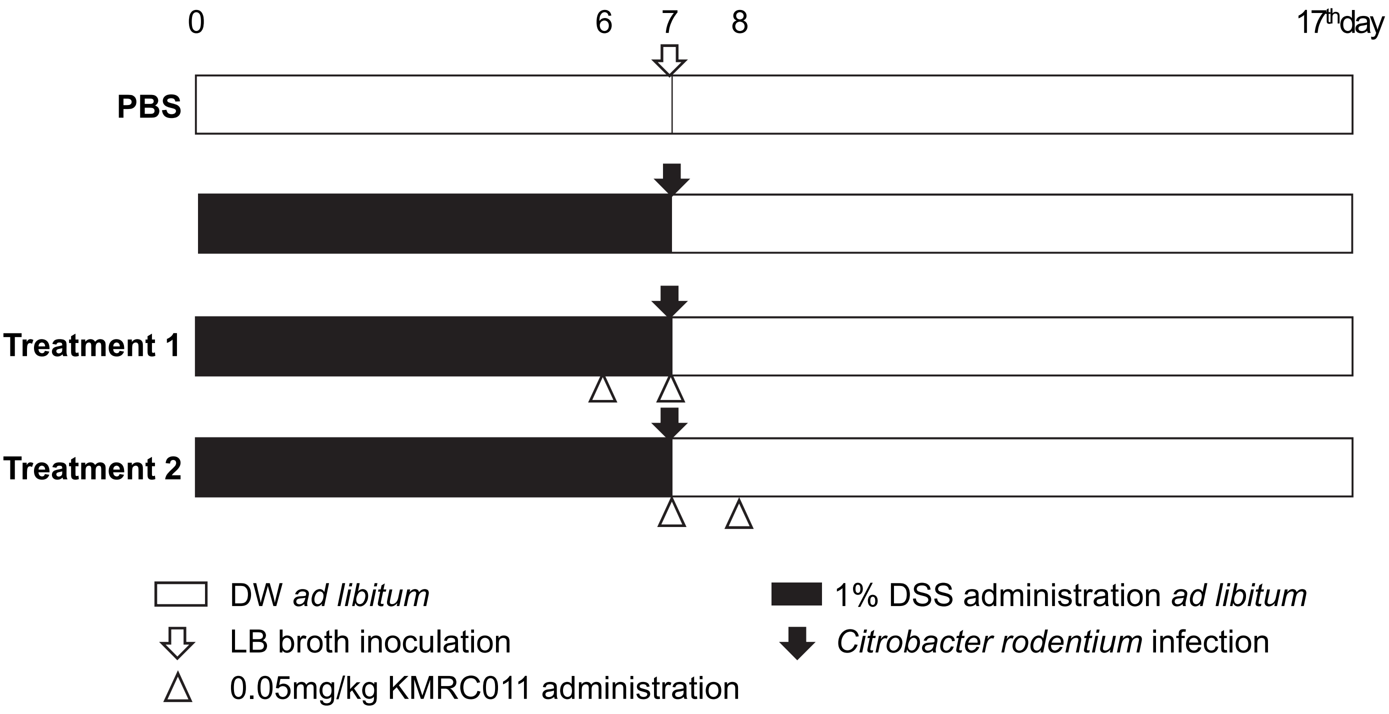

Supplemental Figure S1. Experimental protocol for ulcerative colitis. C57BL/6N mice were divided into 4 groups (each group n=6) and pretreated with 1% DSS or DW for 7 days. Mice were infected with *Citrobacter rodentium* (1.6 x 10^8 CFU) via oral gavage on the 7th day after onset of experiment. Treatment 1 and 2 groups were received KMRC011 before or after *C. rodentium* infection. All mice were sacrificed 17th day after onset of experiment.
